# Exploring Patient Preferences Related to Shared Decision-Making in Chronic Disease Management

**DOI:** 10.7759/cureus.70214

**Published:** 2024-09-25

**Authors:** Turki M Alanzi, Nouf Alanzi, Aisha Majrabi, Ahlam S Alhajri, Lujain Alzahrani, Noura Alqahtani, Abdullah Alqadhibi, Saud Alenazi, Hatim Alsaedi, Eidhah Alghamdi, Norah Bin Hamad, Walaa Habib, Nawal H Alharthi, Maher Alharbi, Nafad N Alyahya

**Affiliations:** 1 Department of Health Information Management and Technology, College of Public Health, Imam Abdulrahman Bin Faisal University, Dammam, SAU; 2 Clinical Laboratory Sciences, Jouf University, Jouf, SAU; 3 Clinical Pharmacy, Brighton University, Brighton, GBR; 4 College of Agricultural and Food Sciences, King Faisal University, Al Hofuf, SAU; 5 Faculty of Medicine, King Abdulaziz University, Jeddah, SAU; 6 College of Pharmacy, King Khalid University, Abha, SAU; 7 Expanded Programme on Immunization (EPI), Ministry of Health, Riyadh, SAU; 8 ‏Transfusion Medicine Services Department, King Abdulaziz Medical City, Riyadh, SAU; 9 Medicine, Taibah University, Madinah, SAU; 10 Medicine, Al Qara General Hospital, Al Baha, SAU; 11 College of Medicine, King Saud University, Riyadh, SAU; 12 Medicine, King Fahad University Hospital, Al Khobar, SAU; 13 Public Health College, Saudi Electronic University, Dammam, SAU; 14 Madinah Health Cluster, Ministry of Health, Madinah, SAU; 15 College of Medicine, Imam Muhammad Bin Saud Islamic University, Riyadh, SAU

**Keywords:** chronic disease management, decision aids, health literacy, patient preferences, shared decision-making

## Abstract

Background and objective

Shared decision-making (SDM) in healthcare has transitioned from a paternalistic model to a collaborative approach, particularly significant in chronic disease management. This shift focuses on aligning healthcare decisions with patient preferences and values, thereby enhancing patient engagement and treatment adherence. However, patient preferences regarding involvement in SDM vary widely, influenced by demographic, disease-specific, psychological, cultural, and social factors. This study aimed to explore patient preferences related to SDM in chronic disease management in Saudi Arabia, by assessing attitudes toward SDM, the impact of decision aids, and the role of clinician communication in influencing these preferences.

Methods

A cross-sectional survey design was employed, involving 409 adult outpatients with chronic diseases attending four public hospitals in Saudi Arabia. Participants were selected using purposive and convenience sampling. The survey, translated into Arabic, collected demographic data and information on preferences and experiences in decision-making, communication, and information sharing. The data were analyzed using SPSS Statistics (IBM Corp., Armonk, NY) to identify patterns and correlations.

Results

Key findings indicated a strong preference among the participants for involvement in treatment decisions (n=303, 74.2%) and clear communication using layman's terms (n=338, 82.6%). Major barriers to active participation in SDM included lack of time during appointments (n=275, 67.2%), difficulty understanding medical terminology (n=220, 53.9%), and feeling intimidated to ask questions (297, 72.6%). Comfort in SDM was highest in the age group of 41-50 years [mean=4.16, standard deviation (SD)=28.44; F=2.3287, p=0.0739]. Patient satisfaction was significantly higher in the age group of 18-30 years (mean=3.42, SD=1.09; F=3.0503, p=0.0284).

Conclusions

Our findings highlight the need for incorporating patient preferences into chronic disease management strategies to enhance engagement and satisfaction.

## Introduction

In recent years, there has been a paradigm shift in the field of healthcare from a paternalistic model, where physicians made decisions on behalf of patients, to a more collaborative approach known as shared decision-making (SDM) [1,2]. This transition is particularly significant in the context of chronic disease management, where patients are often required to make frequent, complex decisions regarding their treatment and lifestyle. Chronic diseases such as diabetes, heart disease, and chronic obstructive pulmonary disease (COPD) represent a substantial burden on both patients and healthcare systems globally [3,4]. These conditions typically require long-term management strategies, including lifestyle modifications, medication adherence, and regular monitoring [5]. Given the chronic nature and complexity of these diseases, effective management hinges on the active participation of patients in their own care. SDM is a process by which clinicians and patients work together to make healthcare decisions that align with the patient's preferences, values, and specific health circumstances [6].

The concept of SDM is deeply rooted in the ethical principles of patient autonomy and informed consent [7]. It recognizes that patients have the right to be fully informed about their medical options and to participate actively in decisions about their care. This approach contrasts with the traditional medical model, where healthcare providers are seen as the primary decision-makers. By fostering a collaborative relationship, SDM aims to enhance patient engagement, adherence to treatment plans, and overall quality of care. Several studies [8-15] have highlighted the positive impact of SDM on patient satisfaction and treatment adherence, particularly in chronic disease management. Patients involved in decision-making processes tend to feel more informed about their conditions and are more likely to adhere to treatment plans [8,9]. For instance, Granados-Santiago et al. [10] have emphasized the importance of patient engagement in SDM, linking it to improved health outcomes and a greater sense of empowerment in managing chronic diseases.

Understanding patient preferences for involvement in SDM is crucial for tailoring healthcare delivery to improve patient outcomes and satisfaction. However, the extent to which patients want to engage in decision-making varies widely [16]. Factors influencing patient preferences in SDM include demographic characteristics (age, gender, education level), disease-specific factors (type and severity of the chronic condition), psychological factors (health literacy, self-efficacy, anxiety), and cultural and social determinants [17-25]. For example, older patients or those with lower health literacy may prefer a more passive role, relying on their providers' expertise, whereas younger patients or those with higher education levels might seek a more active role in their healthcare decisions [25].

Despite the recognized benefits of SDM, its implementation in clinical practice, especially in chronic disease management, faces several challenges [26,27]. Barriers such as time constraints during appointments, difficulties in communicating complex medical information, and a lack of training among healthcare providers in facilitating SDM discussions have been identified in previous studies [11,28]. These challenges underscore the need for strategies to overcome obstacles and promote patient-centered care through tailored SDM approaches aligned with patient preferences and values.

SDM fosters an open, transparent dialogue where patients receive comprehensive, tailored information about their diagnosis and treatment options, enabling them to make informed choices that reflect their preferences, values, and lifestyle. Improved communication through SDM also builds trust, ensuring that patients feel heard and respected in their healthcare journey [29,30]. Furthermore, patient experiences and engagement are significantly enhanced through SDM. Patients actively involved in decision-making often report a greater sense of control and empowerment over their health [31-33]. Engaged patients are more likely to adhere to prescribed medications, follow through with recommended lifestyle changes, and attend follow-up appointments [34-36]. Moreover, SDM significantly heightens patient satisfaction, as patients who are part of the decision-making process are more likely to feel respected and valued as partners in their healthcare, leading to better management of chronic conditions and, consequently, improved clinical outcomes [37,38].

This study aims to explore and understand patient preferences related to SDM in chronic disease management. By examining the factors that influence these preferences and identifying the barriers to effective SDM implementation, it seeks to provide insights that can inform strategies to enhance patient-centered care. Ultimately, the goal of this study is to improve the quality of chronic disease management by ensuring that healthcare decisions are aligned with patient preferences and values. Such insights are critical for designing effective interventions and communication strategies that foster patient engagement, adherence to treatment plans, and overall satisfaction with care. Moreover, by identifying barriers to SDM implementation, this study can guide healthcare policy reforms and the integration of decision-support tools in clinical practice. This research aims to contribute to a more patient-centered healthcare system, improving health outcomes and reducing the burden of chronic diseases on patients and healthcare systems alike [39].

## Materials and methods

A cross-sectional survey design was adopted in this study. The selection of different approaches and the procedures adopted are explained in the following sections.

Study setting and participants

This study specifically focused on adult outpatients (aged 18 years or older) with a variety of chronic conditions, including kidney, lung, and heart diseases, as well as diabetes and various forms of cancer (blood, lung, pancreatic, and breast), from four public hospitals in Saudi Arabia. The inclusion criteria were patients diagnosed with one or more of these chronic diseases and actively receiving outpatient care. Patients under the age of 18, those not diagnosed with any of the specified chronic conditions, or were unable to provide informed consent were excluded. During their outpatient appointments, eligible patients were invited to participate, provided with a detailed explanation of the study's purpose and objectives, and, with their explicit consent, asked to complete an online survey at their convenience within four weeks (February to March 2024).

Selection and sampling

Given the requirement to include patients with chronic conditions in the study, the researchers needed to select an easily available sample. Therefore, this study utilized both purposive and convenience sampling procedures, which are commonly employed in similar research [40]. The participants were selected via purposive selection, which involved choosing individuals based on their current conditions that necessitate the use of telepharmacy services. Additionally, convenience sampling was employed to enroll individuals who could be conveniently contacted, specifically from public university hospitals. The sample size estimation was performed using Cochran's formula [41], yielding a value of 383. The subsequent post-hoc power analysis indicated a power of 100%.

Questionnaire design

The survey was designed to gather detailed insights into patient experiences and preferences. It started with participant information, ensuring ethical compliance and confidentiality (Appendices). It was divided into four sections: (i) demographic information: age, gender, education level, chronic disease status, and duration of disease management; (ii) peferences and experiences in decision-making: this section assessed the importance of involvement in treatment decisions, understanding risks and benefits, quality of life, incorporating personal values, family input, receiving information in layman's terms, discussing treatment preferences with healthcare providers, comfort in expressing preferences, challenges in accessing information, influence of personal research, and barriers to participation in decision-making; (iii) communication and information sharing: the frequency of seeking additional information, satisfaction with clarity and relevance of information, preferred communication methods, value of digital health tools, training on digital tools, obstacles in using technology, satisfaction with healthcare provider accessibility, and timeliness of responses; and (iv) shared decision-making experience: this section explores participation in SDM discussions, perceived benefits, influence on treatment adherence, factors contributing to successful SDM, challenges faced, importance of SDM in managing chronic diseases, interest in decision-making aids, and satisfaction with provider involvement in incorporating patient preferences.

A certified translator [42] translated the questionnaire from English to Arabic. The translated questionnaire was verified by two professors from the eHealth department at Imam Abdulrahman Bin Fahd University. Several grammatical modifications were proposed, and the Arabic rendition was subsequently amended to include them. An exploratory study was conducted with a cohort of 12 patients, and subsequent examination was carried out on the gathered data. The Cronbach alpha coefficient was calculated for all items and was found to be greater than 0.7, indicating strong internal consistency and reliability [43].

Data collection

To gather data, a questionnaire survey was created using Google Forms and delivered to patients through emails and social media channels. Out of the initial group of 482 adult patients, 441 expressed their interest in participating in the study. These patients were then provided the survey link via email and various social media platforms. Out of the 441 patients, 409 patients (response rate: 92.7%) completed the survey, and their responses were considered for data analysis. Before engaging in the survey, the patients received comprehensive information regarding the objectives of the study and were afforded the chance to provide informed consent at their outpatient appointments.

Data analysis

SPSS Statistics Version 24 (IBM Corp., Armonk, NY) was employed to analyze the data. Descriptive statistics were used to depict the demographic information of the individuals. Furthermore, the data were analyzed using a two-sample t-test with unequal variances (for analyzing the significant differences in perceptions between gender groups) and a single-factor ANOVA (for analyzing the significant differences in perceptions between age groups and groups based on education levels).

Ethics-related factors

The study was approved by the Research Ethics Committee at Imam Abdulrahman Bin Faisal University. All participants provided informed consent before participating by signing the consent form during the outpatient appointments. Measures were taken to ensure participant confidentiality and data security, including anonymizing responses and storing data on secure servers. The study adhered to all relevant ethical norms, and no conflicts of interest or funding sources were reported, thereby maintaining research integrity and mitigating bias.

## Results

Participant demographics

The survey participants' data (Table 1) revealed a diverse demographic profile, with a significant portion (46.2%) of respondents falling in the 18-30 age group. The gender distribution indicated a predominance of male participants (64.3%) compared to females (35.7%). As for education, most participants had a Bachelor's degree (77.3%).

**Table 1:** Participant demographics

Variable		N	%
Age group, years	18-30	189	46.2%
	31-40	83	20.3%
	41-50	86	21.0%
	51-60	51	12.5%
Gender	Male	263	64.3%
	Female	146	35.7%
Education	Uneducated	35	8.6%
	Primary/secondary education	65	15.9%
	Diploma	57	13.9%
	Bachelor’s degree	216	52.8%
	Master’s degree	36	8.8%

While 197 (48.3%) had been managing their chronic disease for the past one to three years, 77 (18.9%) had been managing it for less than one year; 88 (21.6%) participants for over five years, and 46 (11.2%) for the past three to five years.

Participants’ preferences in SDM

**Figure 1:**
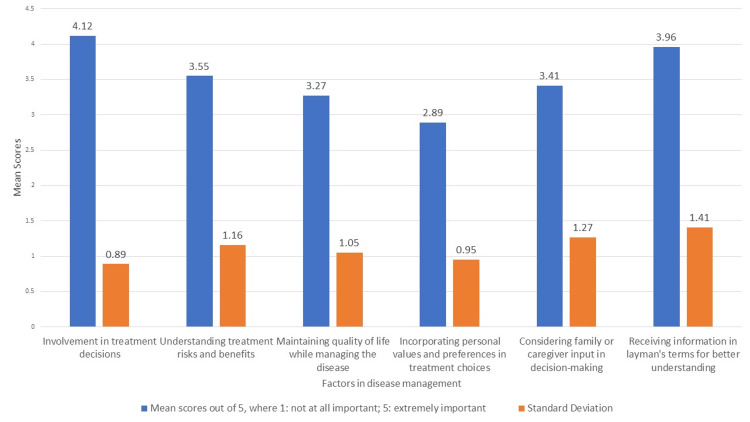
Importance of various factors in managing chronic diseases in SDM SDM: shared decision-making

As shown in Figure 1, the analysis of the mean scores for the importance of various factors in SDM revealed notable participant preferences. The most valued aspect was the active involvement in treatment decisions, indicating that patients highly prioritized being engaged in their treatment planning. Closely following this was the importance of receiving information in a way that is easy to understand, highlighting the need for clear communication. Understanding the risks and benefits of treatment was also important, although slightly less so than direct involvement and clear explanations. The input of family or caregivers in decision-making was recognized as significant, reflecting the supportive role they play in the process. Additionally, there was concern for maintaining quality of life while managing the disease, which underscored the balance sought between treatment and daily living. Lastly, incorporating personal values and preferences in treatment choices, while relevant, was the least prioritized among the factors considered.

One hundred and fifty-nine participants (38.9%) did not discuss their preferences or goals with their healthcare provider, leaving the decision entirely to the provider. Almost half of the participants discussed their preferences only to some extent, while 47 (11.6%) engaged in more extensive discussions. A very small number (n=6, 1.4%) had not yet discussed their preferences but were planning to do so in the future. Furthermore, 256 (62.7%) participants stated that they frequently encountered challenges in accessing information about different treatment options in SDM, while 55 (13.5%) stated they rarely encountered them. The influence of factors such as personal research, online information, or advice from friends/family on the participants' decision-making process was observed to be significant by 212 (51.8%) participants, while 24 (5.9%) denied any influence.

Of note, 321 (78.4%) of the participants believed that it is extremely important to know about alternative treatment options available when discussing with healthcare providers. The barriers to active participation in SDM as experienced by participants included lack of time during appointments (n=275, 67.2%), difficulty understanding medical terminology (n=220, 53.9%), feeling intimidated to ask questions (n=297, 72.6%), and limited access to information/resources (n=112, 27.5%); 266 (65.1%) participants preferred a designated support person (e.g., patient advocate or family member) to be present during discussions on SDM. Furthermore, 342 (83.7%) participants stated that the internet and social media are very influential in decision-making about managing their condition.

Communication and information sharing

A significant majority of the participants (n=359, 87.8%) stated that they frequently sought additional information about their condition and potential treatments beyond the healthcare provider's recommendations.

**Figure 2:**
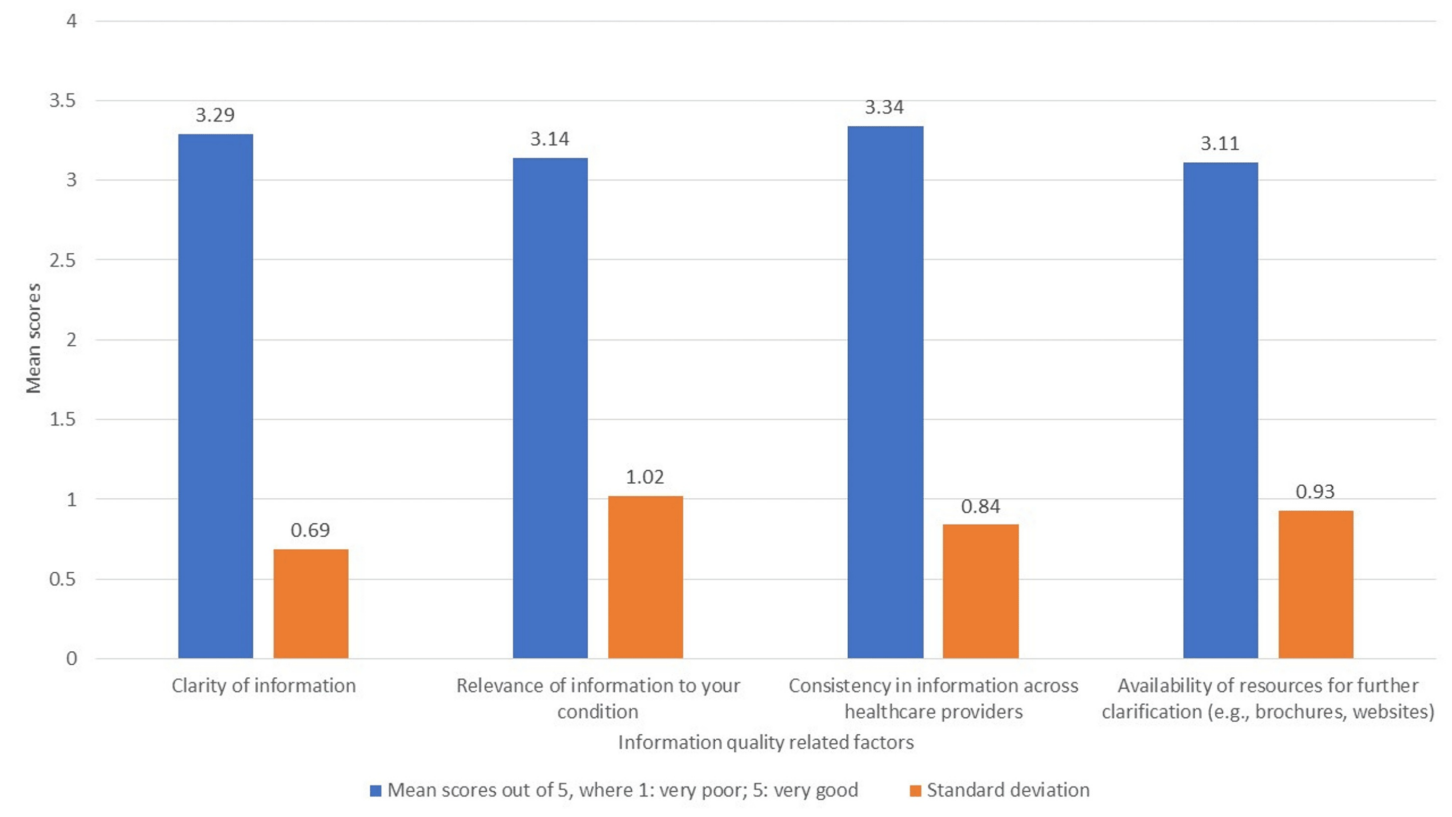
Quality of information from healthcare providers in SDM SDM: shared decision-making

The assessment of information quality attributes provided by healthcare professionals in SDM (Figure 2) indicates moderate patient satisfaction. Patients generally perceive the information from different healthcare providers to be consistent, reflecting a uniformity in communication. The clarity of the information is acknowledged, though there remains scope for enhancement. Regarding the relevance of the information to individual conditions, patients find it moderately applicable. However, there is a notable need for better availability of resources, such as brochures and websites, to offer further clarification and support patient understanding.

Concerning communication preferences, in-person appointments (n=161, 39.5%) hold the highest preference due to the comprehensive care and personal interaction they offer. Phone calls are favored by one-third of participants, for their convenience and immediate communication without requiring physical presence. Email communication (n=63, 15.4%) is valued for its convenience and documentation but is less immediate and personal. Telemedicine/video calls (n=77, 18.9%) are increasingly popular, providing flexibility and remote access, though they are not yet as preferred as in-person visits for all types of care. About three-fourths of the participants observed that digital health tools (apps, wearable devices, etc.) are very valuable in managing their condition and communicating with healthcare providers. However, barriers such as concerns about data privacy/security (n=353, 86.3%) and lack of support or guidance from healthcare providers (n=314, 76.9%) were observed to be prevalent.

SDM experience

Nearly half of the participants occasionally participated in SDM, followed by those who took part regularly (n=80, 19.5%), while 88 (21.6%) did not participate in SDM and left the decision to their healthcare provider; 261 (63.8%) participants strongly agreed that SDM positively influences adherence to the treatment plan and effectively managing their condition.

**Figure 3:**
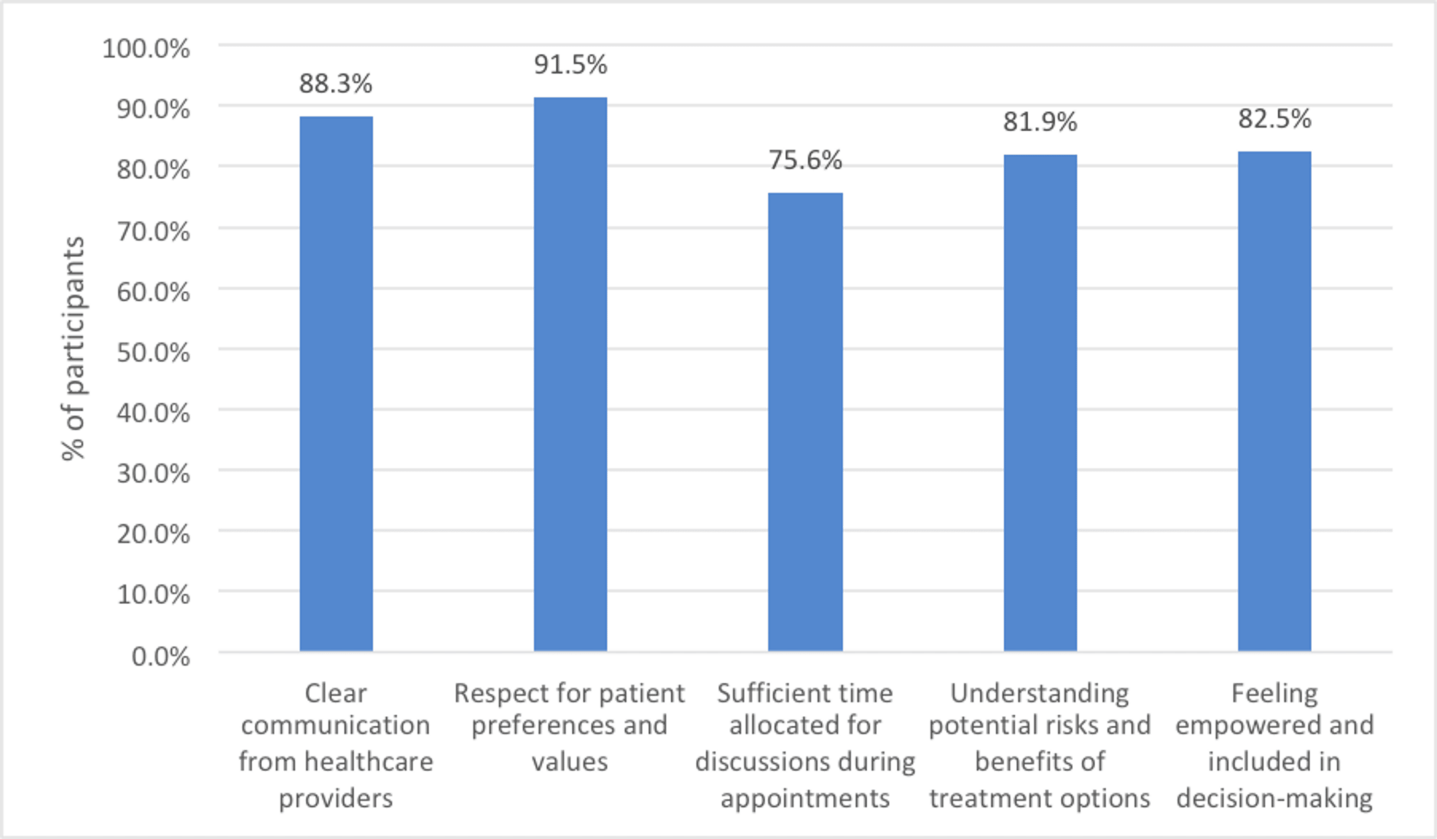
Factors contributing to effective SDM in managing chronic diseases SDM: shared decision-making

The participants' responses regarding factors contributing to effective SDM in managing chronic diseases (Figure 3) highlight several critical areas. Most participants highly value clear communication from healthcare providers, emphasizing its essential role in SDM. The importance of respecting patient preferences and values is also strongly emphasized, showing that personalizing care to align with these factors is crucial. However, there is room for improvement in the allocation of sufficient time for discussions during appointments, as this aspect was rated lower by participants. Understanding the potential risks and benefits of treatment options is recognized as important, though with slightly less emphasis. Lastly, the significance of feeling empowered and included in the decision-making process is highlighted, underscoring the need for patient empowerment and active involvement in their care decisions.

The analysis presented in Table 2 provides several insights. Participants' comfort levels with SDM were relatively consistent across age groups, with the 41-50 age group reporting the highest comfort level. However, there were no statistically significant differences in comfort between age groups. In terms of information quality in SDM, the 41-50 age group also rated this aspect the highest, while the 31-40 age group had the lowest ratings. Again, no significant differences were found across age groups regarding perceptions of information quality. As for the importance of SDM, the 51-60 age group placed the highest importance on it, but there were no significant differences between the age groups as to how important they found SDM. However, in terms of patient satisfaction, the 18-30 age group reported the highest levels of satisfaction, significantly higher than other age groups, indicating a notable difference in how younger patients perceive their satisfaction with SDM.

**Table 2:** Differences in participants' perceptions related to SDM (by age groups) *Statistically significant difference SD: standard deviation; SDM: shared decision-making

Factors	Age group, years	N	Mean	SD	F	P-value
Comfort in SDM	18-30	189	3.32	1.56	2.3287	0.0739
	31-40	83	3.30	1.75		
	41-50	86	4.16	28.44		
	51-60	51	3.24	1.42		
Information quality in SDM	18-30	189	3.25	0.83	2.0345	0.1084
	31-40	83	2.99	1.36		
	41-50	86	3.33	0.92		
	51-60	51	3.31	0.91		
Importance of SDM	18-30	189	2.99	1.31	0.7305	0.5342
	31-40	83	2.98	1.15		
	41-50	86	2.98	1.41		
	51-60	51	3.24	1.14		
Patient satisfaction	18-30	189	3.42	1.09	3.0503	0.0284*
	31-40	83	3.04	1.08		
	41-50	86	3.29	1.08		
	51-60	51	3.12	1.31		

As shown in Table 3, there were significant differences across all factors when the cohort was stratified based on educational levels. Comfort in SDM increases as education levels rise, with uneducated participants reporting the lowest comfort and those with a master’s degree experiencing the highest. Similarly, the quality of information perceived by participants improves with higher education, with those holding a bachelor’s degree rating it the highest. The importance placed on SDM also correlates with education, with more educated participants viewing it as more crucial. Additionally, patient satisfaction follows this pattern, with higher education levels associated with greater satisfaction. These findings suggest that individuals with higher education tend to feel more comfortable, perceive better information quality, place more importance on SDM, and experience greater satisfaction in their healthcare interactions.

**Table 3:** Differences in participants' perceptions related to SDM (by education) *Statistically significant difference SD: standard deviation; SDM: shared decision-making

Factors	Educational levels	N	Mean	SD	F	P-value
Comfort in SDM	Uneducated	35	2.89	1.75	3.2108	0.0129*
	Primary/secondary level	65	3.09	1.99		
	Diploma	57	3.09	1.94		
	Bachelor’s degree	316	3.59	1.18		
	Master’s degree or higher	36	4.75	66.71		
Information quality in SDM	Uneducated	35	2.24	0.80	25.213	<0.0001*
	Primary/secondary level	65	2.81	0.81		
	Diploma	57	2.86	0.99		
	Bachelor’s degree	316	3.57	0.69		
	Master’s degree or higher	36	3.40	0.98		
Importance of SDM	Uneducated	35	2.54	1.08	4.7073	0.0011*
	Primary/secondary level	65	2.80	1.66		
	Diploma	57	2.75	1.33		
	Bachelor’s degree	316	3.20	1.13		
	Master’s degree or higher	36	3.17	1.06		
Patient satisfaction	Uneducated	35	2.60	1.54	17.246	<0.0001*
	Primary/secondary level	65	3.02	1.48		
	Diploma	57	2.65	0.95		
	Bachelor’s degree	316	3.60	0.81		
	Master’s degree or higher	36	3.47	0.54		

As presented in Table 4, there were significant differences between males and females across all factors related to SDM. Males reported feeling more comfortable with SDM compared to females, indicating a higher level of ease in their participation. In medication management, males again scored higher, suggesting greater confidence or involvement in managing their medications. Additionally, males perceived the quality of information in SDM more positively than females. These results highlight a notable gender disparity, with males generally reporting higher levels of comfort, better perceptions of information quality, and more confidence in medication management compared to females.

**Table 4:** Differences in participants' perceptions related to SDM (by gender) *Statistically significant difference SD: standard deviation; SDM: shared decision-making

Factors	Gender	N	Mean	SD	F	P-value
Comfort in SDM	Male	263	3.98	9.81	26.494	<0.0001*
	Female	146	2.59	1.54		
Medication management	Male	263	3.45	0.76	42.106	<0.0001*
	Female	146	2.82	1.11		
Information quality in SDM	Male	263	3.22	1.10	25.301	<0.0001*
	Female	146	2.65	1.39		
Patient satisfaction	Male	263	3.54	0.84	48.287	<0.0001*
	Female	146	2.82	1.32		

Lastly, patient satisfaction was significantly higher among males, with a mean of 3.54 compared to 2.82 for females (F=48.287, p<0.0001). These results demonstrate that males generally have higher comfort, better perceived medication management and information quality, and greater satisfaction in SDM compared to females.

## Discussion

The findings of this study offer critical insights into patient preferences for SDM in the context of chronic disease management. The demographic profile of the participants reveals a predominantly younger, educated, and male cohort, which may influence their preferences and experiences in SDM. Education level significantly influences patient comfort, perceived information quality, importance of SDM, and satisfaction. Patients with bachelor's degrees or higher report greater comfort and satisfaction, emphasizing the need for healthcare providers to tailor their communication and SDM approaches based on the educational background of their patients. This finding aligns with previous research [13-19], highlighting the role of demographic factors in shaping patient preferences for SDM. Age also plays a role, with younger patients showing higher satisfaction levels. This demographic trend suggests that younger patients may have higher expectations for involvement in their healthcare decisions and may benefit more from SDM approaches that actively engage them. It also underscores the importance of tailoring SDM approaches to specific patient populations to enhance engagement and satisfaction.

Patient preferences and factors influencing SDM

The analysis indicates that patients highly value involvement in treatment decisions, emphasizing the necessity for healthcare providers to foster an environment that encourages active patient participation. The preference for receiving information in layman's terms highlights the need for clear and comprehensible communication to facilitate better understanding and informed decision-making. These findings align with previous studies [20,21], underscoring the significance of effective communication in SDM. The variability in patient preferences for SDM, influenced by factors such as age, education level, and duration of disease management, is evident. Older patients or those with lower health literacy tend to prefer a more passive role, relying on their healthcare providers' expertise. In contrast, younger, more educated patients seek a more active role in their healthcare decisions. This variability necessitates a personalized approach to SDM, recognizing that a one-size-fits-all strategy may not be effective.

Challenges and barriers to SDM implementation

Despite the recognized benefits of SDM, the study identifies several barriers to its effective implementation. Time constraints during appointments, difficulties in understanding medical terminology, and a lack of support or guidance from healthcare providers are significant obstacles. These barriers reflect the challenges highlighted in previous research [23,28], indicating a need for strategies to overcome these issues by aligning healthcare decisions with patient values and preferences and training healthcare professionals in SDM. The significant influence of personal research, online information, and advice from friends or family on patient decision-making emphasizes the key role of external sources in SDM. This finding suggests that healthcare providers should acknowledge and incorporate these influences into their discussions to ensure that patients feel heard and respected.

Communication and information sharing

The preference for in-person appointments over other communication methods, despite the growing popularity of telemedicine, indicates that face-to-face interactions remain crucial for effective SDM. This underscores the importance of personal interaction in building trust and ensuring clear communication [44,45], particularly in the context of Saudi Arabia, where healthcare decisions are often influenced by cultural values that emphasize strong patient-provider relationships [28]. Hence, patients may prefer in-person interactions to foster trust, gain reassurance, and ensure their concerns are fully understood. This aligns with the broader cultural emphasis on personal connections and respect for authority figures, such as healthcare providers, making clear communication crucial in SDM. However, the value placed on digital health tools highlights the potential for integrating these technologies to support SDM, provided that concerns about data privacy and security are addressed. The moderate levels of satisfaction with the information quality provided by healthcare providers indicate room for improvement. Ensuring consistency, clarity, and relevance of information, besides providing accessible resources for further clarification, are key steps toward enhancing patient satisfaction and engagement in SDM.

Implications for practice and policy

The insights gained from this study have several implications for clinical practice and healthcare policy. Healthcare providers should adopt a personalized approach to SDM, considering the demographic characteristics and individual preferences of their patients. Training programs for healthcare providers should emphasize the importance of clear communication, understanding patient preferences, and using decision-support tools to facilitate effective SDM. Healthcare policies should support the integration of SDM into clinical practice by addressing the identified barriers, such as time constraints and the need for patient education. Additionally, promoting the use of digital health tools while ensuring data privacy and security can enhance patient engagement and support effective SDM.

Limitations

The study's limitations include a predominantly younger, educated, and male sample, which may not represent the broader chronic disease population. Another limitation is the potential for selection bias, as the online survey may not have reached individuals without internet access or necessary devices, and those with lower educational qualifications. This could impact the generalizability of the findings. The reliance on self-reported data introduces potential biases, affecting the accuracy of findings. Additionally, the cross-sectional design limits the ability to establish causality in the observations. Future research should aim to include more diverse populations and utilize longitudinal designs to better understand causal relationships.

## Conclusions

This study highlights the essential role of understanding patient preferences in achieving effective SDM in chronic disease management. By recognizing the variability in patient preferences and addressing the barriers to SDM, healthcare providers can enhance patient engagement, satisfaction, and adherence to treatment plans. The findings highlight the need for personalized, patient-centered approaches to SDM, supported by clear communication and the integration of digital health tools. Ultimately, this research contributes to a more patient-centered healthcare system, thereby improving health outcomes and reducing the burden of chronic diseases on patients and healthcare systems alike.

## Appendices

**Table 5:** Survey questionnaire

Factors	Variables/items	Options
Demographics	Age group, years	18-30, 31-40, 41-50, 51-60, >60
	Gender	Male/female
	Education	High school or equivalent. Diploma: college or technical training. Bachelor's degree. Master's degree or higher
	Do you have any chronic disease(s) that you manage regularly?	Yes/No
	How long have you been managing your chronic disease(s)?	Less than 1 year. 1-3 years. 3-5 years. More than 5 years
Preferences and experiences in decision-making	Rate the importance of the following in your decision-making process regarding chronic disease management (on a scale of 1 to 5, with 1 being not important and 5 being extremely important): Involvement in treatment decisions. Understanding treatment risks and benefits. Maintaining quality of life while managing the disease. Incorporating personal values and preferences in treatment choices. Considering family or caregiver input in decision-making. Receiving information in layman's terms for better understanding	1 to 5
	Have you discussed your treatment preferences and goals related to your chronic disease(s) with your healthcare provider(s)?	Yes, extensively. Yes, to some extent. No, but I would like to. No, I prefer my healthcare provider to make decisions
	How comfortable do you feel expressing your treatment preferences, concerns, or doubts to your healthcare provider(s)?	Very comfortable. Moderately comfortable. Neutral. Slightly uncomfortable. Very uncomfortable
	In the past, have you encountered challenges in accessing information about different treatment options for managing your chronic disease(s)?	Yes, frequently. Yes, occasionally. No, rarely. No, never
	How much influence do factors such as personal research, online information, or advice from friends/family have on your decision-making regarding chronic disease management?	Significant influence. Moderate influence. Minimal influence. No influence
	Are there specific aspects or types of information that you feel are lacking or insufficient in discussions about your chronic disease(s) and treatment options with your healthcare provider(s)?	Yes. No. Not sure
	How important is it for you to have alternative treatment options available when discussing your chronic disease management with your healthcare provider(s)?	Extremely important. Very important. Moderately important. Slightly important. Not important at all
	What barriers, if any, have you encountered in actively participating in decision-making about your chronic disease management? (Select all that apply) Lack of time during appointments. Difficulty understanding medical terminology. Feeling intimidated to ask questions. Limited access to information/resources. Other (please specify):	
	Would you prefer to have a designated support person (e.g., patient advocate, family member) present during discussions about your chronic disease management with your healthcare provider(s)?	Yes, always. Yes, sometimes. No, never
	How do you perceive the role of the internet and social media in influencing your decisions about managing your chronic disease(s)?	Very influential. Moderately influential. Slightly influential. Not influential
Communication and information sharing	How often do you seek additional information about your chronic condition(s) and potential treatments outside of your healthcare provider's recommendations?	Rarely or never. Occasionally. Frequently. Always
	How satisfied are you with the following aspects of the information provided by your healthcare provider(s) regarding your chronic condition(s) and treatment options? (Rate each on a scale of 1 to 5, with 1 being very dissatisfied and 5 being very satisfied): Clarity of information. Relevance of information to your condition. Consistency in information across healthcare providers. Availability of resources for further clarification (e.g., brochures, websites)	1 to 5
	In what ways would you prefer to communicate with your healthcare provider(s) regarding your chronic condition(s) and treatment options? (Select all that apply): In-person appointments. Phone calls. Email communication. Telemedicine/video calls. Secure messaging through a patient portal. Mobile applications for health monitoring. Other (please specify):	
	How valuable do you find digital health tools (apps, wearable devices, etc.) in managing your chronic disease(s) and tracking your health progress?	Very valuable. Moderately valuable. Slightly valuable. Not valuable at all
	Do you feel adequately trained or informed about using digital health tools for managing your chronic condition(s)?	Yes, completely. Yes, to some extent. No, but I am interested in learning. No, I am not interested
	What obstacles, if any, have you encountered in using technology or digital health tools to manage your chronic disease(s)? (Select all that apply): Limited access to the necessary technology. Difficulty understanding or using the tools. Concerns about data privacy/security. Lack of support or guidance from healthcare providers. Other (please specify):	
	How satisfied are you with the accessibility of your healthcare provider(s) when you have questions or concerns about your chronic condition(s)?	Very satisfied. Moderately satisfied. Neutral. Moderately dissatisfied. Very dissatisfied
	How would you rate the timeliness of responses from your healthcare provider(s) when communicating about your chronic disease(s) and related inquiries?	Very timely. Moderately timely. Neutral. Moderately delayed. Very delayed
Shared decision-making experience	Have you participated in shared decision-making discussions with your healthcare provider(s) regarding your chronic disease management?	Yes, regularly. Yes, occasionally. No, but I would like to. No, I prefer my healthcare provider to make decisions
	What aspects of shared decision-making do you find most beneficial? (Open-ended response)	
	Do you feel that shared decision-making positively influences your adherence to the treatment plan for your chronic condition(s)?	Strongly agree. Agree. Neutral. Disagree. Strongly disagree
	In your opinion, what factors contribute to successful shared decision-making in managing chronic diseases? (Select all that apply): Clear communication from healthcare providers. Respect for patient preferences and values. Sufficient time allocated for discussions during appointments. Understanding potential risks and benefits of treatment options. Feeling empowered and included in decision-making. Other (please specify):	
	Have you ever faced challenges or conflicts in shared decision-making discussions with your healthcare provider(s)? If yes, please describe briefly	
	How important do you consider shared decision-making when coping with changes or adjustments in your chronic disease management plan?	Extremely important. Very important. Moderately important. Slightly important. Not important at all
	Would you be interested in participating in decision-making aids or tools (e.g., decision-making guides, videos) to better understand treatment options for your chronic condition(s)?	Yes, strongly interested. Yes, somewhat interested. No, not interested. Unsure
	How satisfied are you with the involvement of your healthcare provider(s) in incorporating your preferences and values into the treatment plan for your chronic disease(s)?	Very satisfied. Moderately satisfied. Neutral. Moderately dissatisfied. Very dissatisfied
